# Assessing Naltrexone Prescribing and Barriers to Initiation for Alcohol Use Disorder: A Multidisciplinary, Multisite Survey

**DOI:** 10.3389/fpsyt.2022.856938

**Published:** 2022-05-10

**Authors:** Jonathan G. Leung, Prasanna P. Narayanan, Matej Markota, Nathaniel E. Miller, Kemuel L. Philbrick, M. Caroline Burton, Robert W. Kirchoff

**Affiliations:** ^1^Pharmacy Services, Mayo Clinic, Rochester, MN, United States; ^2^Department of Psychiatry and Psychology, Mayo Clinic, Rochester, MN, United States; ^3^Mayo Family Clinic Kasson, Kasson, MN, United States; ^4^Division of Hospital Internal Medicine, Mayo Clinic, Rochester, MN, United States

**Keywords:** alcohol use disorder, naltrexone, prescribing, substance use disorder, survey

## Abstract

**Objective:**

To survey barriers in prescribing naltrexone for alcohol use disorder.

**Methods:**

A 12-question survey related to naltrexone prescribing patterns, perceptions, and knowledge was sent to 770 prescribers in the departments of internal medicine, family medicine, and psychiatry across a health system with sites in Arizona, Florida, and Minnesota.

**Results:**

Responses were obtained and included for 146/770 prescribers (19.0% response rate). Most respondents were in the department of internal medicine (*n* = 94, 64.4%), but the departments of psychiatry (*n* = 22, 15.1%) and family medicine (*n* = 30, 20.5%) were also represented. Only 34 (23.3%) respondents indicated they had prescribed naltrexone in the previous 3 months. The most common reasons for not prescribing naltrexone were “unfamiliarity with naltrexone for treatment of alcohol use disorder” and “patients do not have appropriate follow-up or are not in a formal treatment program.” Compared with those representing internal/family medicine, psychiatry respondents were more likely to prescribe naltrexone and answer knowledge questions correctly.

**Conclusion:**

In this survey among primarily non-addiction-trained prescribers, a disparity was shown for prescribing naltrexone and in knowledge barriers between staff in internal/family medicine and psychiatry. There exist opportunities for education and quality improvement that promote the prescribing of naltrexone for alcohol use disorder by non-addiction specialists.

## Introduction

Among persons with alcohol use disorder (AUD), few will ever receive any form of treatment, including medications for AUD (1, 2). In 2019, it was estimated that of 14.5 million Americans, 7.6% received some type of treatment, and 1.6% received a medication to manage alcohol use (3). Low rates of AUD treatment may result from numerous factors, including treatment ambivalence among patients, stigma, insufficient training and skills of clinicians, and insurance or other administrative barriers within the health care system (4–7). A crucial need exists to understand modifiable factors that interfere with a patient's access to evidence-based AUD treatment, including pharmacotherapy.

Naltrexone is 1 of 3 US Food and Drug Administration (FDA)—approved medications for AUD; acamprosate and disulfiram are the others. Naltrexone is an opioid antagonist that can augment alcohol-induced sedation and other negative alcohol effects, while decreasing hedonic effects and reducing alcohol cravings in persons with AUD (8–10). Patients do not need to be sober before starting naltrexone and do not need to maintain sobriety during naltrexone use. Continued use of alcohol as naltrexone is started may be beneficial through a process known as pharmacologic extinction, i.e., by reducing the association between alcohol consumption and the reinforcing effects experienced (11). Even intermittent or “as needed” use before drinking may confer benefits (12, 13).

Overall, naltrexone has a low monitoring burden, few contraindications, and is well tolerated (14–16). Naltrexone can also be administered as a long-acting intramuscular injection that may confer adherence benefits. Clinical barriers to prescribing naltrexone for AUD may include the risk of hepatotoxicity and a need for patients to be opioid free for a minimum of 7 to 10 days before the drug is begun (17). Other described barriers are related to prescribers' knowledge and perception of naltrexone treatment for AUD (5, 18). To better understand the potential barriers to prescribing naltrexone, a multidisciplinary survey was conducted across 3 sites of a large academic medical enterprise.

## Methods

The project was deemed exempt by the Mayo Clinic Institutional Review Board. All relevant data supporting the findings of this study are reported within the article.

Prior survey data have shown that naltrexone is the most commonly prescribed medication for AUD; therefore, we created a survey focused solely on naltrexone (19–21). J.G.L. and R.W.K reviewed other published related surveys, clinical guidelines, and prescribing information for naltrexone that were available through the National Library of Medicine DailyMed and then developed a 12-component electronic survey that explored practitioners' perceptions and knowledge of naltrexone (17, 19–22). A survey draft was reviewed by coinvestigators from the departments of internal medicine, family medicine, psychiatry, and pharmacy. The survey was then reviewed and approved by 3 research committees from the departments of internal medicine, psychiatry, and pharmacy.

The Mayo Clinic Survey Research Center (SRC) created the electronic survey and distributed the link to potential respondents. The SRC also aided in survey readability, design, and structure. A reminder email was sent 3 and 6 weeks after the survey was distributed, and the survey closed 12 weeks after the initial distribution. Data from respondents were deidentified and pooled by the SRC to ensure anonymity. Survey completion by respondents was voluntary, anonymous, and without incentive or remuneration. The survey was distributed to 770 possible respondents in internal medicine physicians, family medicine physicians, psychiatrists, and advanced practice providers (APPs), i.e., nurse practitioners and physician assistants, at 3 practice sites in Arizona, Florida, and Minnesota. Of the 770 surveys, 149 (19.4%) were sent to Arizona, 95 (12.3%) to Florida, and 526 (68.3%) to Minnesota.

The survey included questions about the respondent's years in practice, whether the respondent had additional addiction psychiatry/medicine training, and their percent of time spent working in an outpatient setting, inpatient setting, or nondirect patient care activities. For the purpose of categorization, a respondent's primary clinical practice setting was defined as the clinical practice area (i.e., inpatient or outpatient) with the greatest percentage of time.

Respondents were asked to recall for the prior 3 months the percentage of patients treated for AUD or secondary sequelae, whether they had prescribed naltrexone for AUD, and how many patients they prescribed naltrexone for AUD. For those who had not prescribed naltrexone, an additional question was asked about barriers to prescribing naltrexone for AUD. The survey also contained questions about the respondent's perceptions of naltrexone (e.g., burden, tolerability, and benefits in AUD). Knowledge questions assessed adverse events, drug interactions, clinical monitoring, and appropriate time for initiation based on AUD treatment guidelines. Questions asked are listed in Tables 1–**3**.

**Table 1:** Demographic information and prescribing patterns.

	**Respondents**		
**Questions**	**All (*N* = 146)**	**Internal medicine/family medicine (*n* = 124)**	**Psychiatry (*n* = 22)**
How many years have you been in practice? Mean (SD) (*n* = 134)	13.9 (10.5)	13.6 (9.9)	15.8 (13.2)
Within the last 3 months, approximately what percentage of your time was spent in inpatient, outpatient, and non-direct patient care? No. (%) (*n* = 135)	Inpatient: 59 (43.7) Outpatient: 75 (55.6) *All non-direct patient care 1 (0.7)*	Inpatient: 49 (43) Outpatient: 64 (56.1) *All non-direct patient care: 1 (0.9)* (*n* = 114)	Inpatient: 10 (47.6) Outpatient: 11 (52.4) (*n* = 21)
Have you had any additional training in addiction psychiatry/medicine? No. (%) (*n* = 135)	Yes: 14 (10.4)	Yes: 10 (8.1)	Yes: 4 (18.1)
Within the last 3 months, what percentage of patients whom you cared for were admitted to the hospital or seen in the clinic for alcohol use disorder–related concerns, including secondary sequelae? Mean (SD) (*n* = 137)	9.9% (15.4%)	6.6% (11.7%)	26.8% (20.8%)
Have you prescribed naltrexone treatment for a patient with alcohol use disorder in the last 3 months? No. (%) (*n* = 142)	Yes: 34 (23.9)	Yes: 24 (19.3)	Yes: 10 (45.5)
If naltrexone was prescribed: How many patients have you prescribed naltrexone for in the last 3 months for alcohol use disorder? Mean (SD) (*n* = 33)	2.4 (2.8)	1.8 (1.7)	3.9 (4.4)

### Statistical Analyses

Answers were summarized using descriptive statistics, and differences between psychiatric and non-psychiatric (i.e., internal medicine and family medicine) prescribers were assessed using the Pearson χ^2^ test. In the case of a low number of responses (i.e., <5) to a particular question option(s), the Fisher exact test was used to assess for differences among the categorical data. A *P* value of <0.05 was considered significant.

## Results

After the survey was closed, 146 persons had responded to the survey request, for a response rate of 19.0% (Table 1). Included in the results were respondents from all 3 of the Mayo Clinic campuses: Arizona (*n* = 25, 17.1%), Florida (*n* = 9, 6.2%), and Minnesota (*n* = 112, 76.7%). More physicians responded (*n* = 93, 63.7%) than APPs (*n* = 53, 36.3%). Most respondents were in the department of internal medicine (*n* = 96, 65.8%), but the departments of psychiatry (*n* = 33, 22.6%) and family medicine (*n* = 17, 17.1%) were also represented. Of those responding to percent of time spent in clinical practice, 75 (56%) spent more time in an outpatient setting, and 59 (44%) spent more time in an inpatient setting. Of respondents who answered the question related to additional training in addiction (*n* = 135), 10 (7.4%) were internal medicine/family medicine prescribers and 4 (3%) were psychiatry prescribers. Thus, 8.8% of internal medicine/family medicine prescribers (*n* = 124) and 18.2% of psychiatry prescribers had additional training in addiction medicine or addiction psychiatry.

We asked the question, “What percentage of patients that you cared for were admitted to the hospital or seen in the clinic for AUD-related concerns, including secondary sequelae?” Of 137 respondents, the mean (SD) was 9.9% (15.4%): inpatient respondents, 14.5% (15.7%); and outpatient respondents, 7.1% (15.8%). Of all respondents, 34 (23.9%) indicated that they had prescribed naltrexone in the previous 3 months to 2.4 (2.8) patients. The rate for prescribing naltrexone was similar for respondents practicing primarily in an inpatient setting (20.3%) vs. an outpatient setting (28%).

Those who responded “no” (*n* = 108) to prescribing naltrexone were asked “why” based on branching logic embedded in the survey. Numerous options were available, including a free-text option, and respondents could select more than 1 reason. The responses are summarized in Table 2. The 2 most common reasons were “unfamiliarity with naltrexone for treatment of AUD” (*n* = 57, 52.8%) and “patients do not have appropriate follow-up or are not in a formal treatment program” (*n* = 40, 37%).

**Table 2:** Reasons for not prescribing naltrexone (*n* = 108)^a^.

**Reason**	**No. (%)**
Unfamiliar with naltrexone for the treatment of alcohol use disorder	57 (52.8)
Patients do not have appropriate follow-up or are not in a formal treatment program	40 (37.0)
Do not treat patients who would benefit from naltrexone for alcohol use disorder	32 (29.6)
Should only be started by an addiction specialist	15 (13.9)
Patients refused/not interested	10 (9.3)
Other^b^	9 (8.3)
Have concerns about the overall efficacy	7 (6.5)
Prefer other agents for alcohol use disorder	6 (5.6)
Should only be started in the outpatient/ambulatory setting	4 (3.7)
Too expensive	1 (0.9)

*^a^Respondents could select more than 1 answer (n = 108).*

*^b^Not the patient's primary prescriber (n = 3), already seen by specialist directing care (n = 3), “I do not want to” (verbatim comment, n = 1), concerns with adherence (n = 1), response not decipherable (n = 1)*.

All respondents were asked knowledge questions and about their perceptions of naltrexone treatment for effectiveness, safety, and monitoring burden. They were also asked if prescribing the medication should be limited to those in addiction medicine or psychiatry. One of the questions asked participants to respond to whether naltrexone was a first-line pharmacologic agent for the management of AUD, and 48 (34%) correctly agreed with this statement. The prescribing information describes naltrexone as contraindicated in the setting of “acute hepatitis or liver failure,” but this question was answered correctly by only one-third of respondents (46 [32.6%]). For the following 2 statements, we looked for negative responses (i.e., the answer of “no” was the correct response), which were answered correctly only about half of the time: “Patients and prescribers must register with an FDA Risk Evaluation and Mitigation Strategy program” (59 [42.1%]); and “Naltrexone is dangerous for persons drinking alcohol,” which received the largest negative response (78 [55.7%]). Ninety-one (69.5%) of those responding selected the correct choice for the pharmacologic mechanism of action for naltrexone. We also asked, “Which of the following is true regarding the long-acting intramuscular injection formulation of naltrexone?” The correct (of 4 possible) answers was chosen by 61 (48.0%) of respondents.

In a secondary analysis, we explored how answering correctly might differ between those who did or did not prescribe naltrexone in the prior 3 months. Of 33 prescribers, 24 (72.7%) correctly answered that naltrexone was a first-line agent for AUD compared with 23 of 107 non-prescribers (21.5%) (*P* < 0.001). Similar results were shown for the questions related to naltrexone being contraindicated in acute hepatitis or liver failure (21 of 33 prescribers [63.6%] vs. 25 of 107 non-prescribers [23.4%] correctly answering; *P* < 0.001); the need for prescribers to register with the Risk Evaluation and Mitigation Strategy program (28 of 33 prescribers [84.8%] vs. 30 of 106 non-prescribers [28.3%] correctly answering; *P* < 0.001); naltrexone mechanism of action (29 of 33 prescribers [87.9%] vs. 61 of 97 non-prescribers [62.9%] correctly answering; *P* < 0.007); and lack of danger related to drinking alcohol while taking naltrexone (31 of 33 prescribers [93.9%] vs. 46 of 106 [43.4%] non-prescribers correctly answering; *P* < 0.001). Similar percentages of respondents did not answer the question about the long-acting intramuscular formulation correctly (13 of 30 prescribers [43.3%] vs. 48 of 96 non-prescribers [50%]).

We compared perception and knowledge questions between internal medicine/family medicine prescribers and psychiatry prescribers. Significant differences between the 2 groups were shown for several questions (Table 3).

**Table 3:** Perception and knowledge responses.

	**No. (%)** ^**a**^			
	**All**	**Internal medicine/family medicine (*n* = 119)**	**Psychiatry (*n* = 21)**	**Internal/family medicine vs. psychiatry responses**
**Perception**				
Naltrexone is effective in assisting patients to stop or reduce drinking (*n* = 140)				*P =* 0.004^b^
Agree Neither agree nor disagree Disagree	96 (68.6) 42 (30.0) 2 (1.4)	78 (65.5) 41 (34.5) 0 (0)	18 (85.7) 1 (4.8) 2 (9.5)	
Naltrexone is effective in reducing alcohol cravings (*n* = 140)				*P* = 0.051^b^
Agree Neither agree nor disagree Disagree	94 (67.1) 43 (30.7) 3 (2.1)	79 (66.4) 39 (32.7) 1 (0.8)	15 (71.4) 4 (19.0) 2 (9.5)	
Naltrexone has a high laboratory monitoring burden (*n* = 139)				*P* = 0.002^b^
Agree Neither agree nor disagree Disagree	4 (2.9) 50 (36.0) 85 (61.1)	3 (2.5) 49 (41.5) 66 (55.9) (n=118)	1 (4.8) 1 (4.8) 19 (90.5)	
Naltrexone should only be prescribed to patients being followed up by a psychiatry or addiction specialist (*n* = 140)				*P* = 0.16^b^
Agree Neither agree nor disagree Disagree	34 (24.3) 37 (26.4) 69 (49.3)	32 (26.9) 32 (26.9) 55 (46.2)	2 (9.5) 5 (23.8) 14 (66.7)	
Naltrexone is safe to initiate in the hospital setting (*n* = 140)				*P* = 0.30^b^
Agree Neither agree nor disagree Disagree	90 (64.3) 45 (32.1) 5 (3.6)	73 (61.3) 41 (34.5) 5 (4.2)	17 (80.9) 4 (19.1) 0 (0)	
Naltrexone has a high adverse effect burden (*n* = 139)				*P* = 0.005^b^
Agree Neither agree nor disagree Disagree	3 (2.2) 70 (50.4) 66 (47.5)	3 (2.5) 67 (56.8) 48 (40.7) (*n* = 118)	0 (0) 3 (14.3) 18 (85.7)	
**Knowledge**				
Treatment guidelines for alcohol use disorder recommend naltrexone as a first-line pharmacologic option (*n* = 141)	Correct: 48 (34.0)	Correct: 34 (28.3) (*n* = 120)	Correct: 14 (66.7)	*P* <0.001^c^
Naltrexone is contraindicated for patients with acute hepatitis or liver failure (*n* = 141)	Correct: 46 (32.6)	Correct: 30 (25.0) (*n* = 120)	Correct: 16 (76.2)	*P* <0.001^c^
Patients and prescribers must register with an FDA Risk Evaluation and Mitigation Strategy program (“no” response) (*n* = 140)	Correct: 59 (42.1)	Correct: 40 (33.6)	Correct: 19 (90.5)	*P* <0.001^c^
It is dangerous for a patient to drink while taking naltrexone (“no” response) (*n* = 140)	Correct: 78 (55.7)	Correct: 59 (49.6)	Correct: 19 (90.5)	*P* <0.001^b^
What is naltrexone's primary mechanism of action in alcohol use disorder? (*n* = 131)	Correct: 91 (69.5)	Correct: 73 (66.4) (*n* = 110)	Correct: 18 (85.7)	*P* = 0.12^b^
Targets and restores gamma-aminobutyric acid and glutamate activities that are disrupted in alcohol use disorder Blocks the effect of endogenous opioids in the brain's reward pathway (correct response) Blocks alcohol's metabolism to acetaldehyde, resulting in physical symptoms such as flushing, nausea, and vomiting Blocks the benzodiazepine receptor in the central nervous system, preventing alcohol from directly binding				
Which of the following is true regarding the long-acting intramuscular injection formulation of naltrexone? (*n* = 127)	Correct: 61 (48.0)	Correct: 47 (43.9) (*n* = 107)	Correct: 14 (70.0) (*n* = 20)	*P* = 0.03
It can be initiated without establishing tolerability to the oral formulation It should not be initiated if the patient has had exposure to opioids within the last 30 days Wallet cards or other identification indicating they are prescribed this medication should be carried by the patient (correct response) It may be administered by self-injection				

*FDA, US Food and Drug Administration.*

*^a^Unless otherwise indicated.*

*^b^Fisher exact test.*

*^c^χ^2^ test*.

## Discussion

This survey assessed prescribing patterns and knowledge about naltrexone among internal medicine, family medicine, and psychiatry prescribers across a multistate health system. Whereas some prescribers did have additional training in addiction medicine or addiction psychiatry, to our knowledge, this survey is the first to focus mainly on non-addiction specialists' perceptions and knowledge of naltrexone. Similar studies have been published, but they focused on prescribers who were members of the American Society of Addiction Medicine or the American Academy of Addiction Psychiatrists (19–21). The outreach to non-addiction medicine specialists is important, given the numbers of patients with AUD in primary care and general psychiatry practices. This rationale is also supported by the Combined Pharmacotherapies and Behavioral Interventions (COMBINE) Study, which showed significantly improved outcomes in AUD management when naltrexone was prescribed by non-specialist practitioners (23).

Patients are more likely to interface with a primary care prescriber, so there is greater opportunity for the identification and treatment of AUD in these practices. Prescribers in primary care also do not need to acquire specialized training/certification to issue naltrexone prescriptions for AUD or to refer patients to a specialty clinic for prescriptions. This underscores the importance of studies such as this one by suggesting that with appropriate education and prescriber-level intervention, access to naltrexone could be increased. However, even with referrals, patients may not follow up with prescribers specifically trained in addiction medicine or addiction psychiatry for various reasons, including costs, access, societal stigma, and patients' unwillingness to recognize the need for AUD-specific treatment. From this survey, we learned that primary care providers were distinctly uncomfortable with prescribing naltrexone. Although only 9.5% of psychiatry prescribers agreed (66.7% disagreed) that “naltrexone should only be prescribed by psychiatry or addiction specialists,” 26.9% of internal/family medicine prescribers agreed (46.2% disagreed) with this statement. In addition, although the majority of both groups agreed that “naltrexone is safe to initiate in the hospital setting,” internal/family medicine prescribers were more likely to agree that naltrexone has high adverse effect and laboratory monitoring burdens. Internal/family medicine prescribers were less likely to agree (65.5%) that “naltrexone is effective in assisting patients to stop or reduce drinking” than psychiatry prescribers (85.7%). These results suggest a large barrier to naltrexone use may be primary care provider comfort with and knowledge of the drug.

It is not surprising that psychiatry respondents reported higher naltrexone prescribing rates than the family/internal medicine respondents. On the basis of identified barriers to naltrexone prescribing, this survey thus suggests an opportunity for education and quality initiatives. Our study found that those who do not prescribe naltrexone were less likely to answer knowledge questions correctly. Through education, prescribers may gain the knowledge they need to increase the number of prescriptions they give patients, which was suggested in a previous survey study published in 2020 (19). That study (19) replicated the design of a previous report that assessed the knowledge and experience of naltrexone prescribing among addiction specialists (21). The authors reported that practitioners with a higher level of knowledge and familiarity with naltrexone were more likely to prescribe it, further highlighting lack of knowledge and comfort as a barrier to prescribing naltrexone. In another study, Wei et al. (24) described a discharge planning protocol that was implemented by training internal medicine residents to use a treatment algorithm specific to patients admitted with AUD. As a result of the protocol, the rates of prescribing naltrexone increased from 0% to 64% (*P* < 0.001), and all-cause 30-day readmission rates were reduced from 23.4 to 8.2% (*P* = 0.04).

The current survey had several limitations. First, recall bias may have affected the accuracy of respondents' answers to questions such as the number of patients with AUD who were prescribed naltrexone or cared for in AUD-related admissions/clinic visits. Second, respondents' answers may not necessarily have been reflective of their practice, representing a disparity between knowledge and practice. Third, whereas survey respondents were from 3 states, all were from a single health system enterprise. Thus, limitations on external validity must be considered because findings may reflect the enterprise clinical practice and not be indicative of national prescribing, perception, or knowledge of naltrexone among health care practitioners. Fourth, the overall response rate was low, at 19%, making statistical comparisons between specialists less robust, increasing potential response bias, and introducing type-II error. Fewer psychiatrists responded, so caution is needed in interpreting a statistically significant effect as reflecting real practice. Additional study is needed to determine the underlying causes for this detected distinction between psychiatry and internal/family medicine. Finally, 23.9% of respondents indicated that they had prescribed naltrexone in the prior 3 months. This percentage is higher than that in a recent report from the Substance Abuse and Mental Health Services Administration in which <2% of patients received any medication for managing AUD (3). Therefore, our findings may not be representative of real-world practice. However, our data are comparable to those of Ehrie et al. who reported that 26.8% of board certified psychiatry and and 17.8% of addiction psychiatry respondents had prescribed either oral naltrexone or long-acting naltrexone (19). Despite these limitations, we hope this study draws attention to the topic.

## Conclusion

From this survey, we showed gaps in knowledge, familiarity with, and prescribing patterns for naltrexone among mostly non-addiction-trained prescribers across the departments of internal medicine, family medicine, and psychiatry across a large hospital enterprise. Thus, an opportunity exists to educate prescribers and to promote prescribing naltrexone for medication-assisted treatment of AUD. Future research that seeks solutions to overcome the barriers associated with naltrexone use is needed.

## Data Availability Statement

The original contributions presented in the study are included in the article/supplementary material, further inquiries can be directed to the corresponding author.

## Ethics Statement

Ethical approval was not provided for this study on human participants because the project was deemed exempt. Written informed consent for participation was not required for this study in accordance with the national legislation and the institutional requirements.

## Author Contributions

All authors listed have made a substantial, direct, and intellectual contribution to the work and approved it for publication.

## Conflict of Interest

The authors declare that the research was conducted in the absence of any commercial or financial relationships that could be construed as a potential conflict of interest.

## Publisher's Note

All claims expressed in this article are solely those of the authors and do not necessarily represent those of their affiliated organizations, or those of the publisher, the editors and the reviewers. Any product that may be evaluated in this article, or claim that may be made by its manufacturer, is not guaranteed or endorsed by the publisher.

## Abbreviations

APPs: advanced practice providers
AUD: alcohol use disorder
FDA: US Food and Drug Administration
SRC: Mayo Clinic Survey Research Center.

## References

[1] Cohen E Feinn R Arias A Kranzler HR. Alcohol treatment utilization: findings from the national epidemiologic survey on alcohol and related conditions. Drug Alcohol Depend. (2007) 86:214–21. 10.1016/j.drugalcdep.2006.06.008 16919401

[2] Harris AH Kivlahan DR Bowe T Humphreys KN. Pharmacotherapy of alcohol use disorders in the veterans health administration. Psychiatr Serv. (2010) 61:392–8. 10.1176/ps.2010.61.4.392 20360279

[3] Substance Abuse and Mental Health Services Administration. Key Substance Use and Mental Health Indicators in the United States: Results from the 2019 National Survey on Drug Use and Health. Rockville. Center for Behavioral Health Statistics and Quality, Substance Abuse and Mental Health Services Administration (2020).

[4] Hagedorn HJ Wisdom JP Gerould H. Implementing alcohol use disorder pharmacotherapy in primary care settings: a qualitative analysis of provider-identified barriers and impact on implementation outcomes. Addict Sci Clin Pract. (2019) 14:24. 10.1186/s13722-019-0151-7 31291996 PMC6617941

[5] Williams EC Achtmeyer CE Young JP. Barriers to and facilitators of alcohol use disorder pharmacotherapy in primary care: a qualitative study in five VA clinics. J Gen Intern Med. (2018) 33:258–67. 10.1007/s11606-017-4202-z 29086341 PMC5834954

[6] Abraham AJ Andrews CM Harris SJ Friedmann PD. Availability of medications for the treatment of alcohol and opioid use disorder in the USA. Neurotherapeutics. (2020) 17:55–69. 10.1007/s13311-019-00814-4 31907876 PMC7007488

[7] Oser ML McKellar J Moos BS Moos RH. Changes in ambivalence mediate the relation between entering treatment and change in alcohol use and problems. Addict Behav. (2010) 35:367–9. 10.1016/j.addbeh.2009.10.024 19926399

[8] Ray LA Green R Roche DJO Magill M Bujarski S. Naltrexone effects on subjective responses to alcohol in the human laboratory: a systematic review and meta-analysis. Addict Biol. (2019) 24:1138–52. 10.1111/adb.12747 31148304 PMC6819195

[9] Helstrom AW Blow FC Slaymaker V Kranzler HR Leong S Oslin D. Reductions in alcohol craving following naltrexone treatment for heavy drinking. Alcohol Alcohol. (2016) 51:562–6. 10.1093/alcalc/agw038 27402770

[10] Lee YK Park SW Kim YK. Effects of naltrexone on the ethanol-induced changes in the rat central dopaminergic system. Alcohol Alcohol. (2005) 40:297–301. 10.1093/alcalc/agh163 15897221

[11] Sinclair JD. Evidence about the use of naltrexone and for different ways of using it in the treatment of alcoholism. Alcohol Alcohol. (2001) 36:2–10. 10.1093/alcalc/36.1.2 11139409

[12] Kranzler HR Tennen H Penta C Bohn MJ. Targeted naltrexone treatment of early problem drinkers. Addict Behav. (1997) 22:431–6. 10.1016/S0306-4603(96)00064-0 9183513

[13] Bold KW Fucito LM Corbin WR. Daily relations among affect, urge, targeted naltrexone, and alcohol use in young adults. Exp Clin Psychopharmacol. (2016) 24:367–75. 10.1037/pha0000090 27690505 PMC5111359

[14] Aboujaoude E Salame WO. Naltrexone: a pan-addiction treatment? CNS Drugs. (2016) 30:719–33. 10.1007/s40263-016-0373-0 27401883

[15] Yoon G Kim SW Thuras P Westermeyer J. Safety, tolerability, and feasibility of high-dose naltrexone in alcohol dependence: an open-label study. Hum Psychopharmacol. (2011) 26:125–32. 10.1002/hup.1183 21437991

[16] Pettinati HM O'Brien CP Rabinowitz AR. The status of naltrexone in the treatment of alcohol dependence: specific effects on heavy drinking. J Clin Psychopharmacol. (2006) 26:610–25. 10.1097/01.jcp.0000245566.52401.20 17110818

[17] DailyMed National National Institutes of Health. Naltrexone Hydrochloride Tablet, Film Coated. National Institutes of Health (2021).

[18] Kirchoff RW Mohammed NM McHugh J. Naltrexone initiation in the inpatient setting for alcohol use disorder: a systematic review of clinical outcomes. Mayo Clin Proc Innov Qual Outcomes. (2021) 5:495–501. 10.1016/j.mayocpiqo.2021.01.013 33997645 PMC8105524

[19] Ehrie J Hartwell EE Morris PE Mark TL Kranzler HR. Survey of addiction specialists' use of medications to treat alcohol use disorder. Front Psychiatry. (2020) 11:47. 10.3389/fpsyt.2020.00047 32116860 PMC7034336

[20] Thomas CP Wallack SS Lee S McCarty D Swift R. Research to practice: adoption of naltrexone in alcoholism treatment. J Subst Abuse Treat. (2003) 24:1–11. 10.1016/S0740-5472(02)00319-7 12646325

[21] Mark TL Kranzler HR Song X. Understanding US addiction physicians' low rate of naltrexone prescription. Drug Alcohol Depend. (2003) 71:219–28. 10.1016/S0376-8716(03)00134-0 12957340

[22] The ASAM National Practice Guideline for the treatment of opioid use disorder: 2020 focused update. J Addict Med. (2020) 14(2S Suppl. 1):1–91. 10.1097/ADM.0000000000000633 32511106

[23] Anton RF O'Malley SS Ciraulo DA Cisler RA Couper D Donovan DM. Combined pharmacotherapies and behavioral interventions for alcohol dependence: the COMBINE study: a randomized controlled trial. JAMA. (2006) 295:2003–17. 10.1001/jama.295.17.2003 16670409

[24] Wei J Defries T Lozada M Young N Huen W Tulsky J. An inpatient treatment and discharge planning protocol for alcohol dependence: efficacy in reducing 30-day readmissions and emergency department visits. J Gen Intern Med. (2015) 30:365–70. 10.1007/s11606-014-2968-9 25092008 PMC4351284

